# Global Inequalities in Female Breast Cancer Burden by Decomposition and Age-Period-Cohort Analysis, 1990–2021

**DOI:** 10.1245/s10434-026-19798-5

**Published:** 2026-07-28

**Authors:** Ke Li, Yiyin Zhang, Zeye Liu, Shu Wang

**Affiliations:** 1 Department of Breast Center, Peking University People’s Hospital, Peking University, Beijing, China; 2 Department of Cardiac Surgery, Peking University People’s Hospital, Peking University, Beijing, China

**Keywords:** Breast cancer, Age-period-cohort, Cross-country inequality analysis, Disability-adjusted life years, Mortality

## Abstract

**Purpose:**

Our objective was to analyze temporal trends and geographic disparities in the burden of female breast cancer from 1990 to 2021, focusing on age, period, cohort effects, and socioeconomic inequalities.

**Methods:**

Using data from the 2021 Global Burden of Disease (GBD) study, we quantified disability-adjusted life years (DALYs) and deaths. Analyses included socio-demographic index (SDI) stratification, temporal decomposition, and an age–period–cohort (APC) model to estimate net drifts. The slope index of inequality (SII) and the concentration index were determined to measure cross-country inequalities, and a Bayesian APC model was used to predict the burden in 2050.

**Results:**

From 1990 to 2021, the DALYs and deaths associated with breast cancer increased worldwide, largely because of population growth and aging. Countries with high SDI values had the highest absolute burden, especially among older women, whereas burdens increased faster among younger groups in regions with low or low-middle SDI values. APC models revealed an increased risk in postmenopausal women and period-related decreases in high SDI countries. In contrast, risks increased or plateaued in low SDI regions. Cohort analysis revealed decreasing risks in high SDI countries but increasing risks in younger cohorts elsewhere. Furthermore, inequality shifted, with greater burdens in low SDI countries. Projections indicate continued global increases, especially after 2040.

**Conclusions:**

The global burden of female breast cancer continues to increase with marked regional and age disparities, highlighting the urgent need for region-specific interventions, such as geriatric care in high SDI areas and improved access to screening and treatment in low SDI regions.

**Supplementary Information:**

The online version contains supplementary material available at https://doi.org/10.1245/s10434-026-19798-5.

Breast cancer is still the most common cancer among women worldwide, accounting for 25% of all cancer cases among women.^1^ It is also the most frequently diagnosed cancer in women in 157 countries, and the fourth leading cause of cancer death among women globally.^1^

The main risk factors for breast cancer include various reproductive factors, lifestyle, genetic factors, and environmental factors.^2^ Reproductive components, including early age at menarche, late age at menopause, advanced age at first birth, having fewer children, and shorter duration of breastfeeding,^2^ have been identified as contributing to the onset of breast cancer. Lifestyle factors also play a significant role, such as the use of hormone replacement therapy, the use of oral contraceptives, alcohol consumption, excess body weight, and physical inactivity, also play significant roles in this context.^3^^,^^4^ Although the impact of genetic factors on breast cancer is undeniable, increasing evidence highlights the significant role of the acquired influence of environmental factors. For instance, studies have shown that environmental factors such as exposure to certain chemicals and pollutants can significantly influence the risk of developing breast cancer.^5^ Additionally, specific lifestyle factors, including diet, physical activity level, and alcohol consumption, have been identified as modifiable environmental influences that can affect breast cancer risk.^6^ Therefore, analyzing the specific environmental-related factors is necessary because of regional differences.

Although the global burden of breast cancer remains substantial, treatment strategies have become increasingly diverse and comprehensive. Current options include surgery, chemotherapy, targeted therapies, immunotherapy, endocrine therapy, and radiation therapy, which are often used in combination and tailored to individual tumor characteristics. In particular, early-stage breast cancer is regarded as potentially curable.^7^ With advancements in diagnostic technologies and treatment protocols, the 5-year survival rate for early-stage breast cancer significantly increased and exceeds 90% in many high-income countries.^8^

Therefore, updated data and more comprehensive analyses are essential for exploring additional potential factors that may facilitate the early detection, screening, and timely treatment of breast cancer in women, thereby improving clinical prognoses. This study aims to investigate the global patterns of incidence and mortality rates in female patients with breast cancer, with a focus on analyzing socioeconomic determinants and their underlying age or temporal factors.

## Methods

### Data Source

The 2021 Global Burden of Disease (GBD) study conducted by the Institute for Health Metrics and Evaluation analyzed the impact of 371 diseases and injuries in 204 countries and regions.^9^ This study provided significant methodological updates compared with the 2019 GBD study, with 19,189 additional data sources for disability-adjusted life-years (DALYs) utilized.^9^ By employing standardized methods, the study generated estimates for key health metrics, including prevalence, incidence, mortality, years of life lost, years lived with disability, and DALYs. These approaches ensured that the results were consistent and comparable across different geographic areas and time periods.

### Case Definition

Breast cancer is characterized by the following codes in the International Classification of Diseases, tenth revision (ICD-10): C50-C50.629, C50.8-C50.929, Z12.3-Z12.39, Z80.3, Z85.3, and Z86.000.

### Burden Description

We quantified the burden of female patients with breast cancer from 1990 to 2021 by examining the all-age DALY rate and death rate per 100,000 individuals across 204 countries/territories. Global mapping was used to visualize variations across these regions. The correlation between the socio-demographic index (SDI) and the DALY rate or death rate for female patients with breast cancer across the 204 countries and regions was illustrated through compound line graphs and dumbbell charts, highlighting the disparities in disease burden associated with different socio-economic development levels.

### Socio-demographic Index

The SDI was used to assess regional socioeconomic development. This composite metric integrates per capita income, average years of education, and fertility rates, with values scaled from 0 (lowest development) to 1 (highest development).^9^ Higher SDI scores reflect greater socioeconomic advancement. For analytical purposes, countries were stratified into five tiers: low, low-middle, middle, high-middle, and high SDI regions according to their 2021 index values.

### Age–Period–Cohort Analysis

An age–period–cohort (APC) model was employed to comprehensively analyze the underlying trends in DALY and mortality rates, stratified by age, diagnostic period, and birth cohort. The analytical framework incorporated three dimensions to systematically adjust for potential confounding factors in the outcome risk estimation. Age effects were modelled through longitudinal age curves capturing the natural progression patterns of female breast cancer; period effects were quantified as period-specific relative risks to assess temporal trends in medical advancements and screening efficacy; and cohort effects were expressed as cohort-specific relative risks to evaluate intergenerational mortality variations associated with evolving risk factor exposures.^10^ Moreover, net drift was expressed as the annual percentage change in DALYs and mortality over time, respectively.

### Decomposition Analysis

Decomposition analysis provides a systematic framework to examine factors influencing changes in disease occurrence and death rates over time. In this approach, the observed fluctuations are divided into three main contributors: changes in disease epidemiology, population growth, and changes in age structure.^11^ By assessing the individual impact of each factor, the method elucidates how breast cancer trends in female patients evolved during the research period. This evaluation approach helps determine whether variations in cancer rates are driven more by shifts in disease susceptibility or by changes in population characteristics.

### Cross-Country Inequality Analysis

The slope index of inequality (SII) and concentration index (CI) serve as standardized metrics for assessing absolute and relative socioeconomic disparities in disease burden, respectively.^12^ These indices evaluate inequalities in the distribution of breast cancer across nations. The SII is derived from regression analysis, which correlates a country’s age-standardized rate (ASR) of DALYs and deaths with its relative SDI ranking, which is determined by the midpoint of its population in an SDI-based cumulative distribution. To account for variability, a weighted regression model is employed to test for heteroscedasticity. The CI, on the other hand, is computed by numerically integrating the area under the Lorenz curve, where the cumulative proportion of the ASR of DALYs or deaths is plotted against the population’s cumulative distribution ranked by the SDI.^13^^,^^14^

### Predictive Analysis

A Bayesian APC (BAPC) model with integrated nested Laplace approximations was employed to project future trends in female breast cancer burden; this model simultaneously accounts for age, period, and cohort effects, thereby effectively addressing data gaps and inherent uncertainties.^15^ By providing credible interval estimates, the model offers a more comprehensive understanding of the complex dynamics underlying disease burden and yields more reliable predictions of future trends. The R-package BAPC (0.0.34) was used to implement this approach, and the population from the United Nations World Population Prospects 1950–2100 (2015 Revision) was used for the projected population from 2022 to 2050.

## Results

### Global and National Burden and Temporal Trends of Breast Cancer in Female Patients From 1990 to 2021

**Fig. 1:**
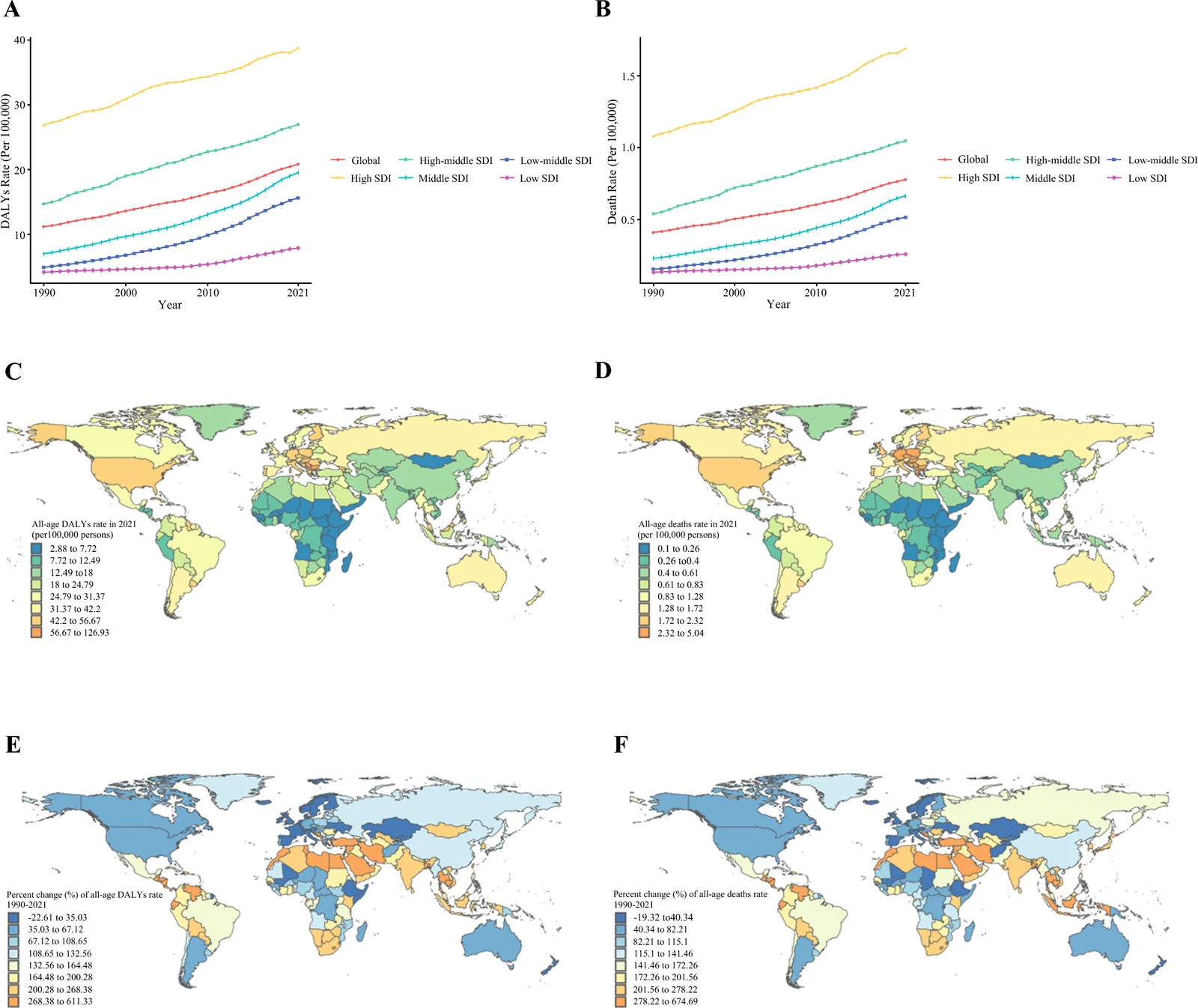
Trends of age-standardized rates of disability-adjusted life-years (DALYs) and mortality of female breast cancer across different socio-demographic index (SDI) regions, 1990–2021. Trends in female breast cancer all-age **A** DALYs and **B** deaths rate. World map of all-age **C** DALYs and **D** deaths for female breast cancer in 2021. **E** World map of percent change of all-age **E** DALYs and **F** deaths for female breast cancer

In 2021, the global DALY for female breast cancer in 2021 was 819,554.62, with an all-age DALY rate of 20.84 per 100,000 people (95% uncertainty interval [UI]: -6.12–49.3), which increased by 9.64 between 1990 and 2021 (Table 1). The worldwide number of deaths in 2021 was 30,568.38, with an all-age death rate of 0.78 per 100,000 people (95% UI: − 0.22–1.84), representing an increase of 0.37 per 100,000 people from 1990 to 2021 (Table 1). The all-age DALY and death rates increased in all five SDI regions. The high SDI region had the highest all-age DALY rate at 38.69 (95% UI: − 11.11–90.14) and the highest all-age death rate at 1.69 (95% UI: − 0.48–3.98) (Table 1, Fig. 1A). Compared with those in 1990, the low-middle SDI region had the greatest increase in the DALY rate (estimated annual percentage [EAPC]: 2.71; 95% confidence interval [CI]: 2.67–2.76) and death rate (EAPC: 2.73; 95% CI: 2.69–2.77) in 2021 (Table 1, Fig. 1B).

**Table 1.** Trends in all-age disability-adjusted life-years (DALYs) and deaths rate across socio-demographic index (SDI) quintiles, 1990−2021

	Global		High SDI		High-middle SDI		Middle SDI		Low-middle SDI		Low SDI	
	1990	2021	1990	2021	1990	2021	1990	2021	1990	2021	1990	2021
*DALYs*												
Number	296,487.61	819,554.62	119,855.12	212,179.34	78,610.67	175,632.44	59,304.57	237,733.86	27,855.25	149,000.51	10,393.71	43,965.54
Percentage of global	100	100	40.43	25.89	26.51	21.43	20	29.01	9.4	18.18	3.51	5.36
All-age rate	11.2 (− 3.27–26.32)	20.84 (− 6.12–49.3)	26.87 (− 7.8–63.03)	38.69 (− 11.11–90.14)	14.71 (-4.34–34.69)	26.96 (− 8.04–62.49)	7.01 (− 2.08–16.72)	19.56 (− 5.65–46.9)	4.89 (− 1.35–11.59)	15.6 (− 4.65–36.97)	4.17 (− 1.18–10.22)	7.88 (− 2.27–18.98)
Percent change of all-age rate, 2019–2021	86.15 (72.22–100.73)		44.03 (33.42–55)		83.23 (64.42–107)		178.99 (146.93–215.38)		219.23 (177.09–271.73)		88.64 (54.62–132.33)	
ASR	13.8 (− 4.02–32.45)	18.05 (− 5.31–42.71)	20.21 (− 5.89–47.72)	20.83 (− 6.04–48.91)	14.17 (− 4.17–33.43)	16.89 (− 5.06–39.16)	10.29 (− 3.03–24.62)	16.24 (− 4.69–38.94)	8.2 (− 2.27–19.45)	18.43 (− 5.45–43.75)	8.29 (− 2.33–20.3)	14.99 (− 4.27–36.32)
Percent change of ASR, 2019–2021	30.82 (21.11–41.13)		3.03 (− 3.75–10.8)		19.2 (6.51–35.13)		57.83 (41.22–77.84)		124.9 (97.6–159.82)		80.71 (50.45–122.7)	
EAPC	0.8 (0.76–0.84)		0.06 (− 0.01–0.13)		0.47 (0.34–0.59)		1.36 (1.29–1.42)		2.71 (2.67–2.76)		1.92 (1.79–2.06)	
*Deaths*												
Number	10,864.81	30,568.38	4809.65	9267.21	2880.74	6816.69	1943.92	8076.8	879.19	4916.36	333.3	1447.82
Percentage of global	100	100	44.27	30.32	26.51	22.3	17.89	26.42	8.09	16.08	3.07	4.74
All-age rate	0.41 (− 0.12–0.97)	0.78 (− 0.22–1.84)	1.08 (− 0.31–2.52)	1.69 (− 0.48–3.98)	0.54 (− 0.16–1.28)	1.05 (− 0.31–2.42)	0.23 (− 0.07–0.55)	0.66 (− 0.19–1.61)	0.15 (− 0.04–0.37)	0.51 (− 0.15–1.23)	0.13 (− 0.04–0.33)	0.26 (− 0.07–0.63)
Percent change of all-age rate, 2019–2021	89.47 (75.73–102.7)		56.76 (43.03–70.23)		94.06 (76.86–114.97)		189.17 (157.45–225.92)		233.72 (194.3–283.98)		93.72 (61.95–137.84)	
ASR	0.52 (− 0.15–1.23)	0.66 (− 0.19–1.57)	0.75 (− 0.22–1.77)	0.78 (− 0.22–1.83)	0.52 (− 0.15–1.24)	0.62 (− 0.18–1.45)	0.37 (− 0.11–0.88)	0.57 (− 0.16–1.37)	0.29 (− 0.08–0.68)	0.65 (− 0.19–1.56)	0.3 (− 0.09–0.74)	0.57 (− 0.16–1.38)
Percent change of ASR, 2019–2021	27.06 (18.38–35.8)		3.68 (− 3.74–11.42)		19.39 (8.67–32.23)		52.4 (36.45–70.94)		125.46 (99.85–157.92)		86.5 (57.63–120.99)	
EAPC	0.72 (0.68–0.76)		0.08 (0.02–0.13)		0.49 (0.34–0.63)		1.26 (1.2–1.32)		2.73 (2.69–2.77)		2.07 (1.94–2.2)	

ASR, age-standardized rate; EAPC, estimated annual percentage change

Data are presented as mean (95% uncertainty interval) unless otherwise specified, except that estimated annual percentage change (EAPC) is expressed as mean (95% confidence interval).

Regionally, the all-age DALY and death rates were highest in Central Europe, whereas the greatest percentage changes in these metrics were observed in North Saharan Africa (Fig. 1C and D), Tropical Latin America and Southeast Asia (Fig. 1E and F).

At the national level, the highest all-age DALY and death rates were observed in the Cook Islands, Palau, Barbados, American Samoa, Niue, Monaco (Fig. 1C and D). Moreover, the greatest increases in the all-age DALY rates and death rates were in Turkey and Egypt, whereas these metrics both decreased in Norway and Burundi (Fig. 1E and F).

Differences in the disease burden between 1990 and 2021 in regions with different SDI values were also evaluated. Globally, the DALY rate for female breast cancer increased across all age groups between 50–59 years and ≥ 80 years (Supplementary Fig. 1A). The most notable increases occurred in the ≥80 age group, followed by women aged 70–79 years. In low SDI regions, increase trends in the DALY rate were observed across all age groups, with the greatest increases among those aged 70–79 years. In high SDI regions, the DALY burden increased sharply among the elderly population, with the most significant change observed in the ≥ 80-year group. The mortality rates followed similar trends (Supplementary Fig. 1B). The death rate increased across all age groups globally, with the sharpest increase among women aged ≥ 80 years. Compared with regions with high SDI values, where mortality increases were less pronounced, regions with low and low-middle SDI values showed substantial increases in death rates over time, especially among elderly people. These findings underscore the increasing burden of breast cancer among older women and highlight disparities in disease outcomes across socio-demographic contexts. The correlations between global and national burdens and SDI levels in 2021 revealed peaks in the DALY and death rates in regions with high–middle SDI values (Supplementary Fig. 2).

### Temporal Trends and APC Effects on DALY and Mortality Rates

**Fig. 2:**
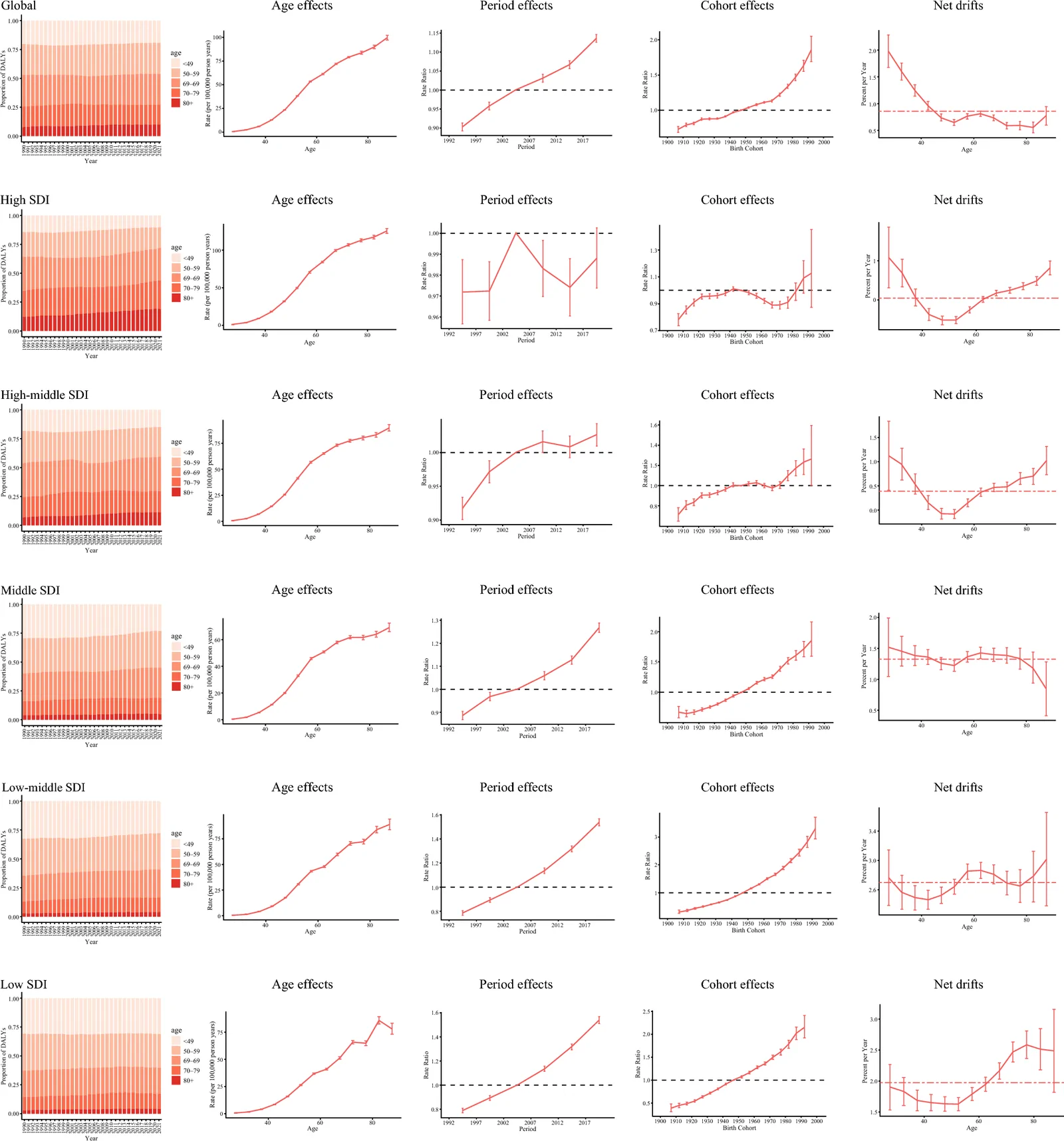
Age-period-cohort effects on all-age disability-adjusted life-years (DALYs) rate across socio-demographic index (SDI) quintiles

**Fig. 3:**
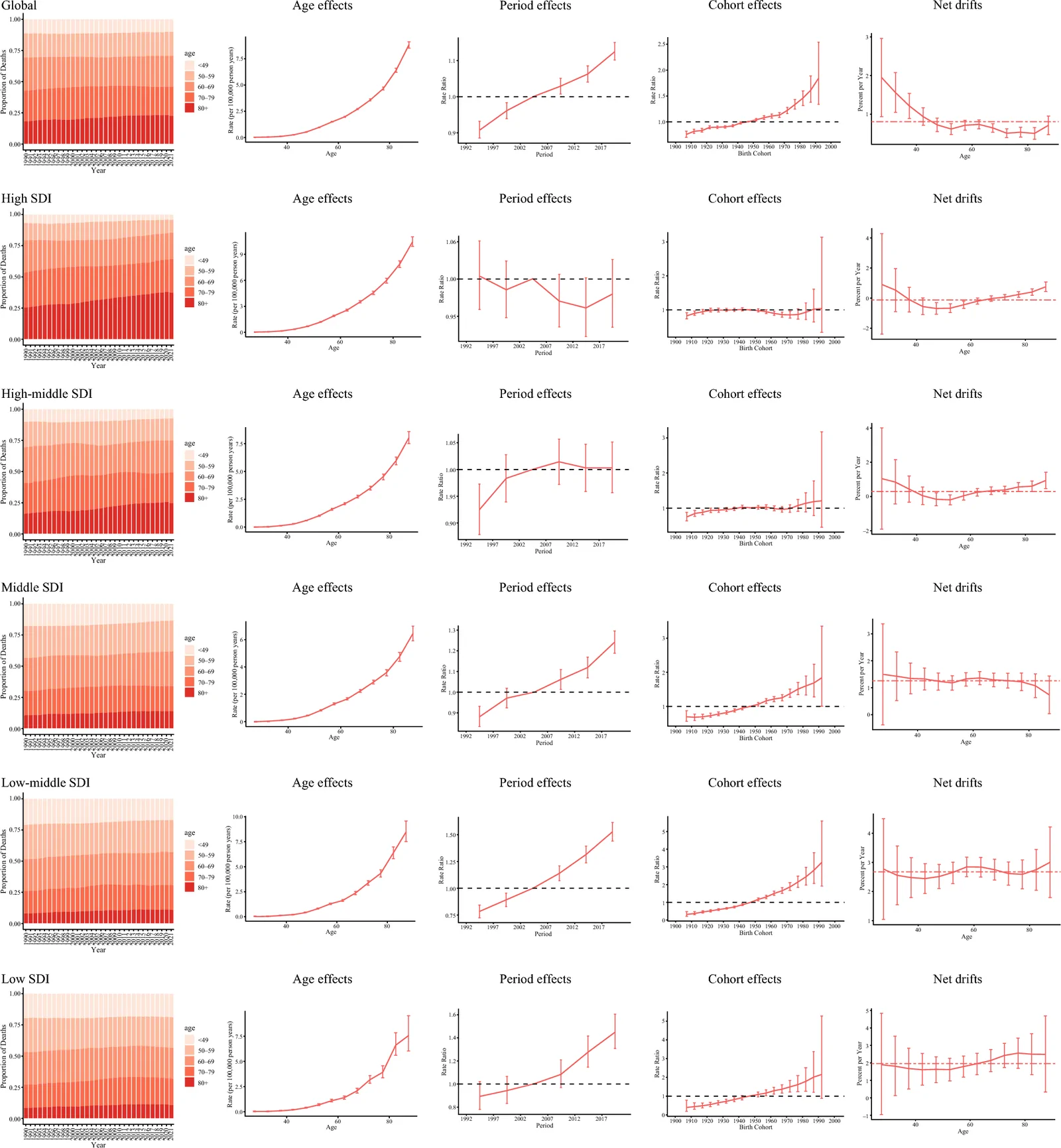
Age-period-cohort effects on all-age deaths rate across socio-demographic index (SDI) quintiles

Age distribution analysis revealed distinct patterns across the different SDI levels. The highest mortality risk among patients with breast cancer aged > 80 years was observed in regions with high SDI values, which progressively increased in recent decades. In contrast, regions with low SDI values presented significantly greater proportional burdens of both DALYs and deaths attributable to early-onset breast cancer in younger populations (Figs. 2 and 3).

Similar patterns in age effects were generally observed across regions with different SDI values, with risk increasing with age. Compared with regions with lower SDI values, the DALY rate among individuals aged > 60 years was greater in countries with high SDI values, suggesting high risks for postmenopausal women. Similarly, the risk of death for these people was greater than that for other age groups and other SDI groups. The second peak in the DALY rate occurred in the low-SDI group for women aged > 80 years.

Similar patterns in age effects were generally observed across regions with different SDI values, with risk increasing with age. Compared with regions with lower SDI values, the DALY rate among individuals aged > 60 years was greater in countries with high SDI values, suggesting high risks for postmenopausal women. Similarly, the risk of death for these people was greater than that for other age groups and other SDI groups. The second peak in the DALY rate occurred in the low-SDI group for women age > 80 years old.

There was a notable regional imbalance of the cohort effects on both DALY and death rates in the past 30 years. Globally, the DALY and death rate ratios consistently increased among younger cohorts, which was accompanied by a progressive increase in disease burden. DALY and death rate ratios consistently exceeding 1 were observed for individuals born after 1940, and the trends in regions with middle, middle–low, and low SDI values were closely aligned with this global pattern. Regions with high and high–middle SDI values exhibited transient declines in DALY and death rate ratios for individuals born around the 1970s, reflecting temporal instability, predominantly among middle-aged and older cohorts.

The results of age-specific net drifts emphasized the decreasing trend of global DALYs and deaths due to female breast cancer, whereas substantial variations were observed across the different SDI levels. In the regions with high and high-middle SDI values, the net drift peaked in early adulthood and during the post-menopause period but decreased to negative values for individuals aged 40–60 years, suggesting a decline in DALY and death burden during this period.

Net drifts were gradually changed across almost all the age groups in regions with middle SDI values, with a slight decline in the oldest ages. Individuals aged approximately 60 years and > 80 years had the highest risks in regions with low-middle SDI values. For regions with low SDI values, moderate net drifts were observed in younger and middle-aged groups, but the sharp increases in older age groups revealed that these individuals had the highest risk of disease burden.

### Decomposition Analysis of Female Breast Cancer Burden Across SDI Regions

**Fig. 4:**
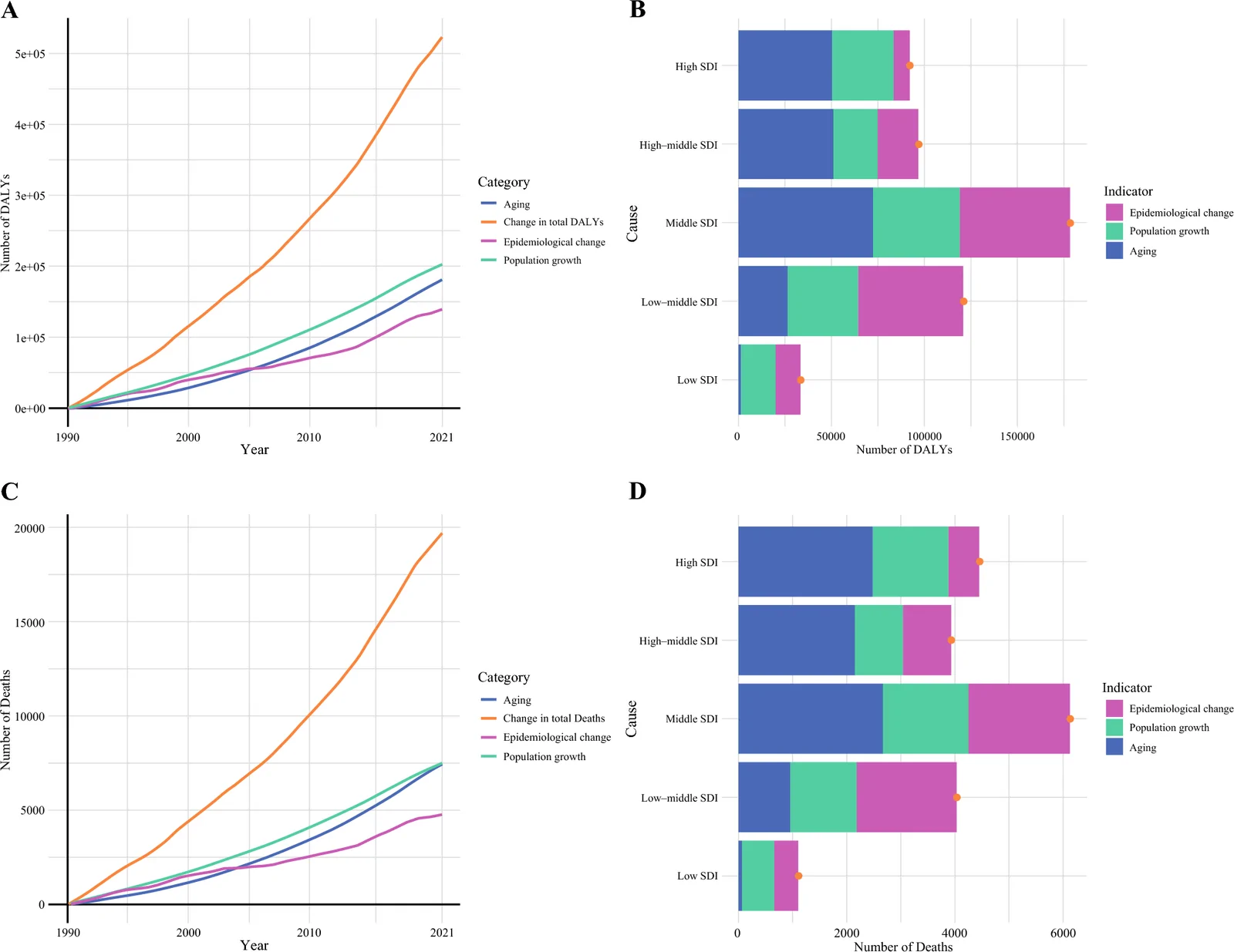
Decomposition analysis. Changes in **A** disability-adjusted life-years (DALYs) and **C** deaths from female breast cancer according to aging, population growth, and epidemiological change from 1990 to 2019 at a global level by socio-demographic index (SDI) quintile. **B** Decomposition analysis for female breast cancer **B** DALYs and **D** deaths

To better understand the factors underlying the increasing burden of female breast cancer, we performed a decomposition analysis of the DALYs and deaths over the past 3 decades. Globally, population growth was the primary contributor to the increase in both DALYs and deaths, followed by age and epidemiological change (Fig. 4A and C). An interesting result was identified in approximately 2005, with aging accounting for the sharpest increase in DALYs and deaths, surpassing the influence of epidemiological change and emerging as the second-most dominant factor, behind population growth.

In 2021, global data stratified by SDI levels revealed substantial heterogeneity in the composition of these drivers. In regions with high and high–middle SDI values, aging contributed most prominently to the increase in both DALYs and deaths, whereas the impact of epidemiological change was minimal. Notably, in regions with low SDI values, aging contributed the least to DALYs and deaths, whereas population growth had the largest impact on the increases in disease burden and mortality (Fig. 4B and D).

### Inequality Analysis of DALYs and Mortality in Female Patients with Breast Cancer

**Fig. 5:**
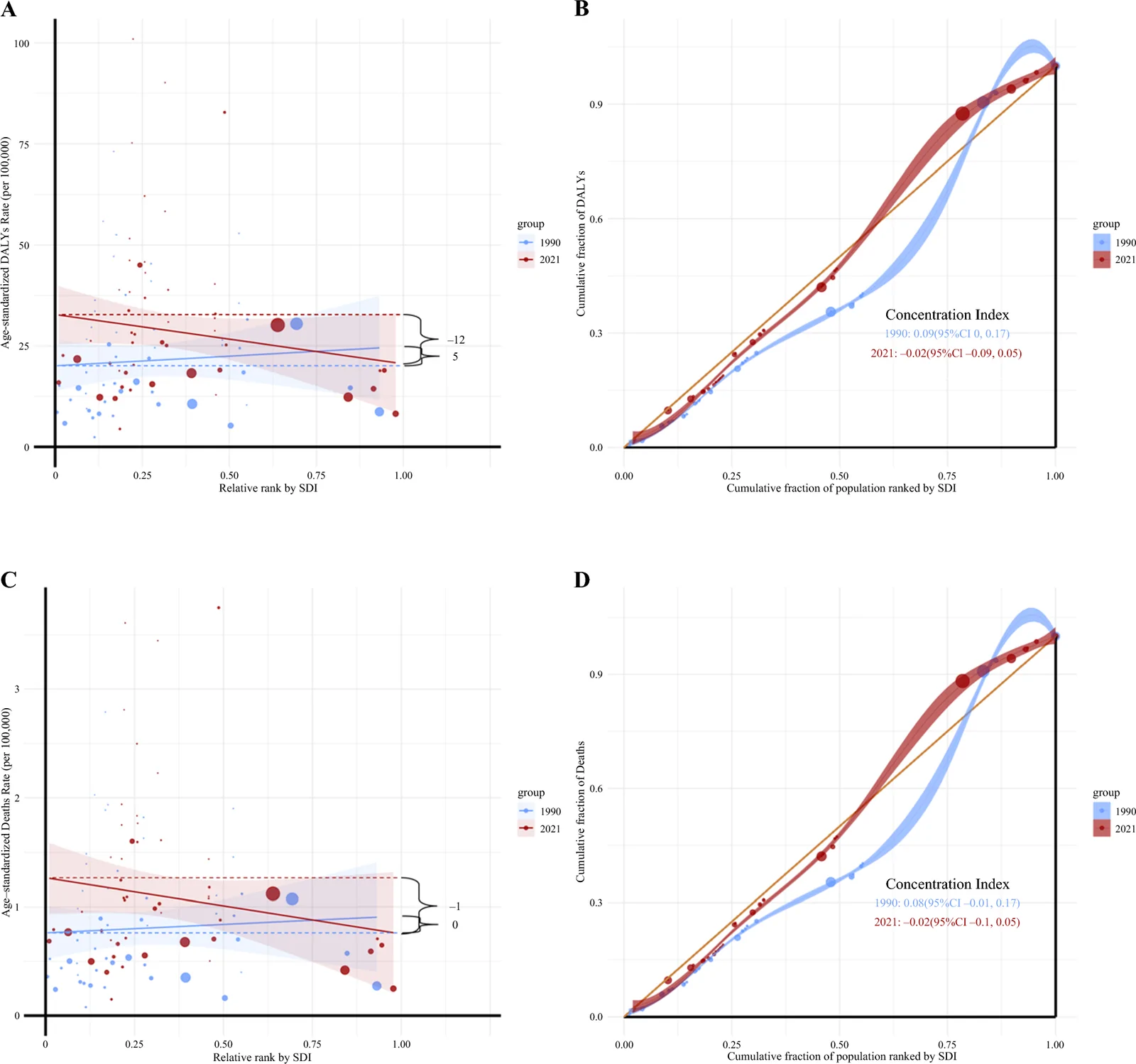
Health inequality regression curves and concentration curves for the prevalence of female breast cancer from 1990 to 2021 across the world. Absolute income-related healthy inequality in age-standardized rate (ASR) of **A** disability-adjusted life-years (DALYs) and **C** deaths burden, presented using regression lines and slope index of inequality (SII), 1990 vs. 2021. **B** Relative income-related healthy inequality in ASR of **B** DALYs and **D** deaths burden, presented using concentration curves and confidence index (CI), 1990 vs. 2021

The SII value of the age-standardized DALY rate shifted from 5 in 1990 to −12 in 2021, reflecting a reversal in health inequality trends over this period (Fig. 5A). Initially, higher SDI groups had more severe DALYs; however, by 2021, the pattern had shifted towards groups with lower socioeconomic status. This negative SII indicates either a relative decline in the health status of historically advantaged populations or a significant increase in disease burden among countries with lower SDI values. Additionally, inequality in countries with SDI values below approximately the 75th percentile improved over time, whereas inequality in countries with higher SDI values may have intensified. The CI results are consistent with the trend in the SII, with the CI value changing from 0.09 (95% CI: 0–0.17) in 1990 to −0.02 (95% CI: -0.09–0.05) in 2021, suggesting an increase in the DALYs among lower-income regions and a gradual deterioration of health outcomes among disadvantaged populations (Fig. 5B).

The SII for the age-standardized death rate also revealed a shift in inequality, with the SII changing from 0 in 1990 to −1 in 2021 (Fig. 5C). This change highlights the increasing mortality burden in low-SDI countries over time. Although there was no marked inequality in 1990, the emergence of a negative SII in 2021 suggests a relative worsening of outcomes for low-income populations. A crossing at approximately the 70th percentile of the SDI indicates that inequality may have increased, particularly in higher SDI countries, although overlapping confidence intervals in the middle SDI range suggest that the overall trend is driven more by worsening outcomes in lower SDI groups. Similarly, the CI changed from 0.08 (95% CI: -0.01–0.17) in 1990 to −0.02 (95% CI: − 0.1–0.05) in 2021, indicating a relative decline in mortality rates among individuals in high SDI countries (Fig. 5D).

### Projection of Female Breast Cancer Burden up to 2050

**Fig. 6:**
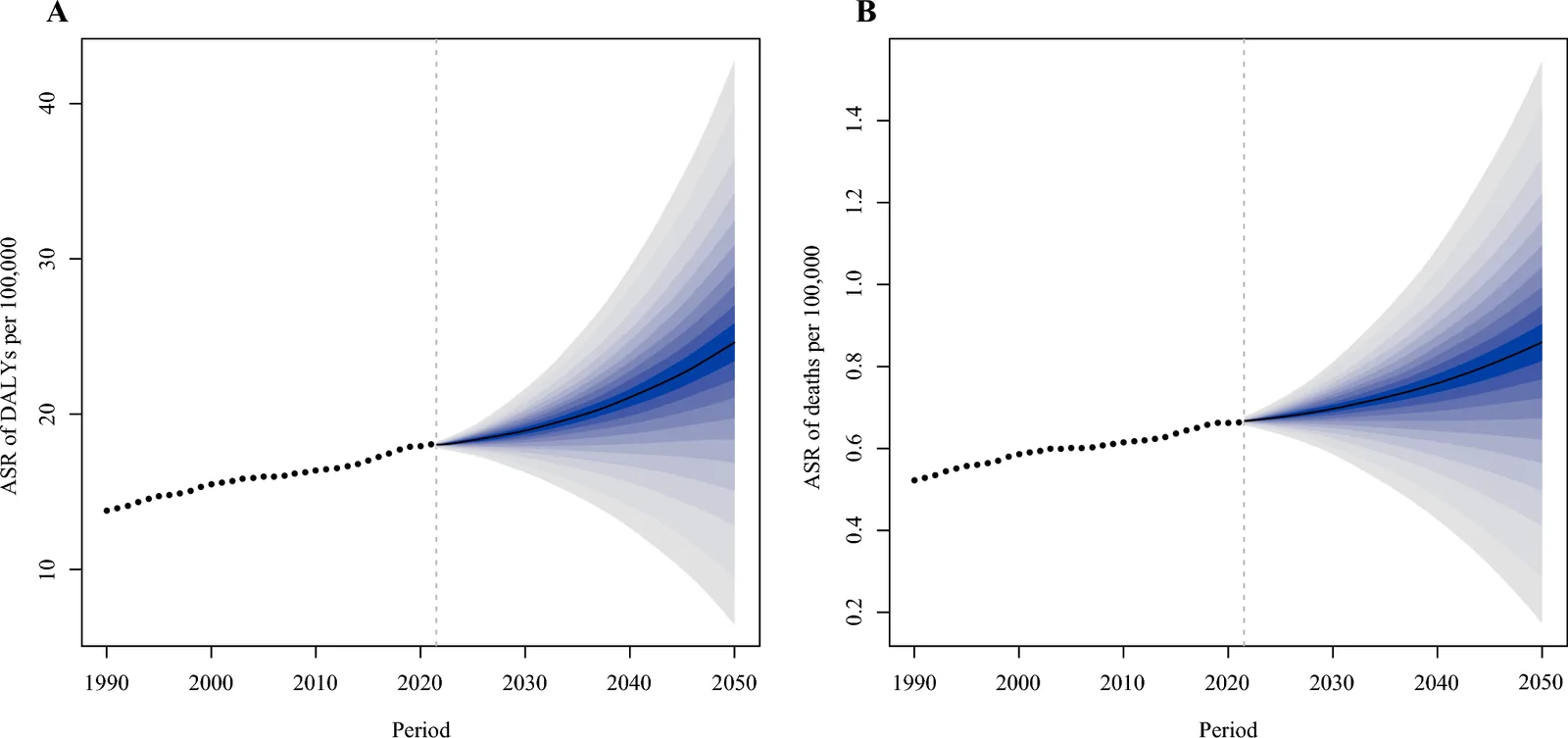
Projects the age-standardized rate (ASR) of disability-adjusted life-years (DALYs) and deaths of female breast cancer to 2050. Predicted ASR of **A** DALYs and **B** deaths to 2050

The projections obtained using the BAPC model are particularly troubling because they predict a continued rise in the burden of female breast cancer through 2050. The ASRs of both DALYs and deaths are predicted to increase annually until 2050; moreover, the growth rates of these metrics are predicted to increase in approximately 2040 (Fig. 6).

## Discussion

In the present study, we explored female breast cancer trends from 1990 to 2021 using the APC model and inequality analyses and revealed complex patterns across different socioeconomic groups. The APC analysis provides a nuanced understanding of how age, diagnostic period, and birth cohort interact to shape the burden of breast cancer, whereas the inequality analysis revealed a concerning reversal in health disparities.

Over the past three decades, the total numbers of female breast cancer DALYs and deaths increased substantially and were accompanied by distinct socioeconomic epidemiological shifts. The most pronounced increase was observed in high-SDI countries, largely driven by demographic shifts toward older populations. The observed peak burden in regions with high–middle SDI values suggest a transitional phase in which risk factor accumulation outpaces healthcare system development. A geographic transition, where the peak absolute burden was observed in Central Europe, while the fastest growth rates were observed in North Africa and Tropical Latin America, was also identified. These patterns collectively signal an evolving global crisis in which gains in high-income settings are increasingly offset by widening disparities in developing regions, demanding differentiated strategies for aging populations and resource-limited health systems. This interpretation is consistent with the latest IARC GLOBOCAN data, which show that female breast cancer remains the most commonly diagnosed cancer and the leading cause of cancer death among women globally, and substantial disparities persist across levels of human development. ^1^^,^^16^ In line with these global trends, our study revealed not only a marked increase in the absolute burden of female breast cancer from 1990 to 2021 but also a shift toward greater concentration of burden in disadvantaged populations.

The APC model elucidated the temporal dynamics and demographic patterns of DALYs and mortality rates in the context of breast cancer. As expected, the age effect revealed that the risk of breast cancer increased significantly with age; this finding aligns with biological susceptibility patterns and the cumulative exposure to risk factors such as hormonal changes and environmental carcinogens in this age group.^17^ In high SDI regions, the breast cancer mortality risk is clearly the highest among women aged >80 years, with a progressive increase in mortality among this population observed in recent decades, This trend likely reflects population aging, increased life expectancy, and possibly suboptimal screening or treatment adherence in older age groups, who often have multiple competing health risks and comorbidities.^17^ Conversely, low SDI regions exhibited substantially greater proportional burdens of both DALYs and deaths that were attributable to early-onset breast cancer in younger populations. This pattern may be driven by several factors. Limited access to timely screening and effective treatment may also lead to later-stage diagnoses, and potential genetic predispositions and environmental influences may disproportionately affect younger women in these regions.^18^ The period effect highlights differential improvements in these metrics across SDI regions. In terms of the period effect, decreasing trends were observed in regions with high and high-middle SDI values, indicating substantial progress in early detection and treatment over time. This improvement was less marked or even absent in low SDI regions, suggesting persistent gaps in public health systems and resource allocation in these areas. Notably, the critical window of 2002–2007 was identified as a turning point across all the SDI groups, potentially reflecting both the delayed implementation of millennium development goals in healthcare systems and the global dissemination of advanced screening technologies during this period.^19^ Birth cohort effects also revealed intriguing patterns. In high-SDI regions, successive birth cohorts exhibited decreasing risks of breast cancer mortality, reflecting the benefits of widespread screening programs. However, in some low and middle SDI countries, cohort effects remained stable or slightly increased.

Globally, population growth accounted for the greatest proportions of increases in DALYs and deaths. This trend was especially prominent in low and middle-income countries undergoing rapid demographic expansion. Moreover, population aging was identified as the second major contributor, particularly in high SDI countries such as Japan and many European nations, where the proportion of older adults is rapidly increasing. As the elderly population grows, the absolute numbers of breast cancer cases and deaths continue to increase.

The reversal in inequality trends, as shown by the change in the SII values from positive to negative, indicates that breast cancer has evolved from a disease that predominantly affects affluent populations to one that increasingly affects disadvantaged groups. The CI results corroborate this trend, revealing an increasing disparity in the disease burden that favors high SDI countries. This may be caused by substantial improvements in high SDI nations through advanced screening programs and comprehensive therapies or through systemic inequities, including critical shortages of oncology resources and specialists in regions with low–middle and low SDI values.^20^ Projections also suggest that current prevention and control strategies will be insufficient to counteract the anticipated increase in breast cancer burden, particularly the accelerated rates predicted after 2040, which likely reflect accumulated exposure to risk factors in aging populations and lagging adaptations of healthcare systems. These findings should be considered in light of the World Health Organization Global Breast Cancer Initiative, which advocates health promotion and early detection, timely diagnosis, and comprehensive breast cancer management as the three pillars for reducing breast cancer mortality worldwide. Accordingly, our results suggest that future control strategies should move beyond general calls for improved cancer control and instead focus on strengthening specific components of the care pathway, particularly in low- and middle-resource settings.

However, our study has several limitations that should be considered. Although the APC model was used to analyze the morbidity of female patients with breast cancer based on the GBD study, it was not a cohort study in the traditional sense; thus, follow-ups in disease and associated risk factors over time are not provided.^10^ Another limitation is related to the use of all-age DALY and death rates. These metrics may be influenced by the varying degrees of aging in different countries within an SDI group. Moreover, we analyzed only data for female breast cancer, but the incidence of male breast cancer is also increasing annually ^21^ and also requires more attention.

## Conclusion

The global burden of female breast cancer continued to increase from 1990 to 2021, with substantial regional, sex, and age disparities. Given the ongoing demographic transitions, the inequitable distribution of the burden of female breast cancer is predicted to remain significant for a long time. Future action for targeted interventions that address the specific needs of different SDI regions to reduce the global burden of breast cancer and mitigate inequalities are urgently needed. High SDI regions may require enhanced geriatric oncology approaches to optimize early detection and management in elderly populations, whereas low SDI regions would benefit from improved screening programs and affordable treatment options targeting younger women.

## Supplementary Information

Below is the link to the electronic supplementary material.

Supplementary file1 (DOCX 13947 kb)

## Data Availability

This manuscript was produced as part of the GBD Collaborator Network and by GBD protocols (contact ID: 0034o00001nHH4NAAW).
