# Supplementary material for: Global Inequalities in Female Breast Cancer Burden by Decomposition and Age-Period-Cohort Analysis, 1990–2021
Source: Ann Surg Oncol. 2026 Jul 28;33(12):10534–46. doi: 10.1245/s10434-026-19798-5 (PMC13675269; doi:10.1245/s10434-026-19798-5)
Supplement: Supplementary file 1 [file 10434_2026_19798_MOESM1_ESM.docx]

**Supplementary Figure 1. Temporal trends in the all-age DALYs and Deaths rate burden from 1990 to 2019.**

(A) DALYs changes at the global level and in the five SDI regions. (B) Changes in the deaths rate at the global level and in the five SDI regions. DALYs, disability-adjusted life years. SDI, socio-demographic index.

**Supplementary Figure 2. The trend of** **ASR of DALYs deaths by SDI from 1990 to 2021 at the global and national levels.**

(A) The trend of ASR of DALYs by SDI from 1990 to 2021 at the global and national levels. (B) The trend of ASR of deaths by SDI from 1990 to 2021 at the global and regional levels. ASR, age-standardized rate. DALYs, disability-adjusted life years. SDI, socio-demographic index.
